# Comprehensive Review of *Cyclamen*: Development, Bioactive Properties, and Therapeutic Applications

**DOI:** 10.3390/ph17070848

**Published:** 2024-06-27

**Authors:** Aya Sharara, Adnan Badran, Akram Hijazi, Ghosoon Albahri, Mikhael Bechelany, Joelle Edward Mesmar, Elias Baydoun

**Affiliations:** 1 Plateforme de Recherche et D’Analyse en Sciences de L’Environnement (EDST-PRASE), Beirut P.O. Box 6573/14, Lebanon; aya.sharara.1@st.ul.edu.lb (A.S.); akram.hijazi@ul.edu.lb (A.H.); g.albahri@st.ul.edu.lb (G.A.); 2 Department of Nutrition, University of Petra, Amman P.O. Box 961343, Jordan; abadran@uop.edu.jo; 3 Institut Européen des Membranes, IEM, UMR-5635, University Montpellier, ENSCM, CNRS, Place Eugene Bataillon, 34095 Montpellier, France; 4 Functional Materials Group, Gulf University for Science and Technology (GUST), Mubarak Al-Abdullah 32093, Kuwait; 5 Department of Biology, American University of Beirut, Beirut P.O. Box 110236, Lebanon; eliasbay@aub.edu.lb

**Keywords:** *Cyclamen*, characteristics, bioactivities, antioxidant, anti-inflammatory, anti-cancerous effects, therapeutic applications, future perspectives

## Abstract

Plants are being researched as potential sources of novel drugs, which has led to a recent acceleration in the discovery of new bioactive compounds. Research on tissue culture technology for the synthesis and processing of plant compounds has skyrocketed, surpassing all expectations. These plants can be bought either raw or as extracts, where some of the chemicals are extracted by mashing the plant in water, alcohol, or another solvent. The use of herbal medicine may open new chances for reducing the onset of infections and treating different diseases including cancer. A perennial plant that blooms in the winter, *Cyclamen*, is one of the most widely used potted flowers in many nations. Alkaloids, flavonoids, phenols, tannins, saponins, sterols, and glycosides are the main active components of *Cyclamen*. Analgesic, cytotoxic, antioxidant, antimicrobial, and anti-inflammatory properties have all been demonstrated as potential effects of various extracts of *Cyclamen* tubers. However, the use of this medicinal plant in official medicine will require further research in the areas of pharmacology. Furthermore, it is necessary to create standard operating procedures for a crude herbal medication. In this regard, this review aims to highlight the key characteristics of the *Cyclamen* plant, such as its various parts, species, stages of development, and geographic range; pinpoint its intriguing bioactivities, its antioxidant, anti-inflammatory, and its anti-cancerous effects; and ascertain its potential medicinal uses and the main future perspectives.

## 1. Introduction

Due to the huge applied studies on plants, the regulation of environmental factors, the induction of gene transfer technologies, and the rise of antibiotic resistance [1], intense attention has been paid to the use of plant extracts for medicinal reasons and the induction of new treatments. It was proposed that herbal medicine (HM) is utilized for preventative purposes or to promote health in addition to treating illnesses [2]. In the last two decades, there has been a considerable rise in the use of herbal medicines, which include medical herbs such as botanical pharmaceuticals, teas, dietary supplements, or indigenous formulations containing herbs [3]. Herb manufacturers are permitted to create, sell, and advertise herbs without first proving their safety and efficacy, as is necessary for pharmaceuticals, under the present legal definition of dietary supplements. Despite the common misconception that herbs are “natural” and therefore safe, a wide range of side effects have been documented due to active ingredients, contaminants, or drug interactions. In traditional medicine, countless plants from all over the world are utilized to cure bacterial infections [4]. Herbal medicines are used by different countries and for different sakes.

Several biological attributes of the *Cyclamen* genus have been documented [5]. Triterpenoid saponins, which are found in *Cyclamen* species, have been proven to have antimicrobial characteristics [6]. Due to their low toxicity, great efficacy, and affordability, saponins are efficient antifungal agents against some fungi, such as the *Candida* genera, which have been the subject of numerous investigations to develop phytotherapeutic treatments for infections. The primary process that creates transmembrane holes compromises the integrity, and results in membrane lysis which appears to be the interaction between the aglycone moieties of the saponins and fungal membrane sterols [7].

The number, type, and sequence of the sugar residues and aglycone portion of the saponins may vary, which could account for differences in their antibacterial capabilities [8,9]. Numerous chemicals, including fatty acids, lycopene, carotenoids, L-theanine, fucoidan, humic acid, sterols, alkaloids, flavonoids, glycosides, saponins, and others are present in the end products [10,11]. The number of recent publications shows a growing interest in medicinal plant research and analysis; from 4686 publications in 2008 to 14,884 publications in 2018, there has been more than a threefold increase in publications. Since the included database records date back to 1800, the output published in just the last eight years of this decade has exceeded all of those combined before 2000 [12].

In this review, we aimed to (1) present major characteristics of the *Cyclamen* plant including its different parts, species, composition, developmental stages, and geographical distribution; (2) identify its potential bioactivities; (3) determine its therapeutic applications; and (4) discuss future perspectives.

## 2. Major Properties of Cyclamen

**Figure 1:**
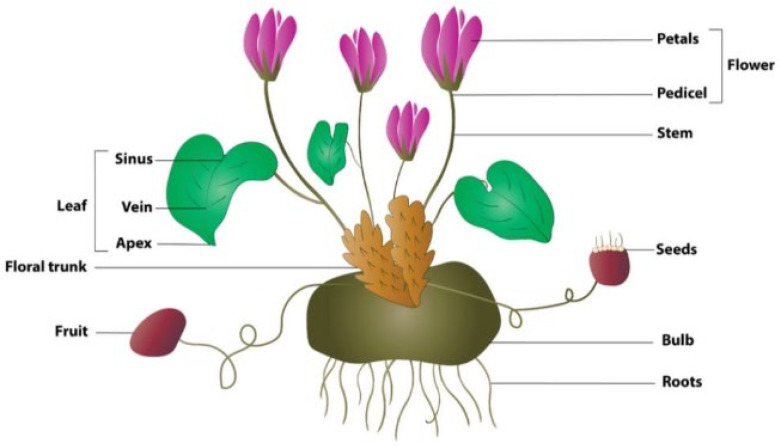
Different parts of the *Cyclamen* plant [17].

Although the development of quality consciousness regarding the evaluation-related evidence is necessary for the efficacy of herbal remedies, meeting consumer demand for botanicals and herbal products is a booming industry [13,14]. Phytotherapy is also an alternative to traditional antidepressants, that may reduce unfavorable side effects [15]. *Cyclamen* is a common and significant pot plant in different countries like Japan, Germany, Italy, The Netherlands, and North America [16]. It is a genus of 22 species of perennial flowering plants consisting of various parts in the family *Primulaceae* (Figure 1).

Every species in this family develops a tuber and can reproduce through seed, although they never do so through natural splitting [18]. Horticulture must generate high and stable seed numbers, and they are pollinated only once per week [19,20]. The main *Cyclamen* species are *C. somalense*, *C. libanoticum*, *C. hederifolium*, *C. persicum* Mill., *C. mirabile* Hildebr., *C. coum* [21]. The clade made up of *Aegiceras*, *Grammadenia*, *Myrsine*, and *Hymenandra* has the closest genetic relationship to Cyclamen, according to phylogenetic analyses of the *Primulaceae*, *Myrsinaceae*, and *Theophyllastaceae* [22]. *Cyclamen somalense* is the first species described in tropical Africa; it is named so due to its locality in Northern Somalia. It has a small 3 cm wide tuber, and from the base of the tuber, the fleshy roots emerge. It looks like *Cyclamen persicum*, however it has elongated tubers, wide, and small leaves with corolla lobes 20–37 × 10^−18^ mm [23].

**Figure 2:**
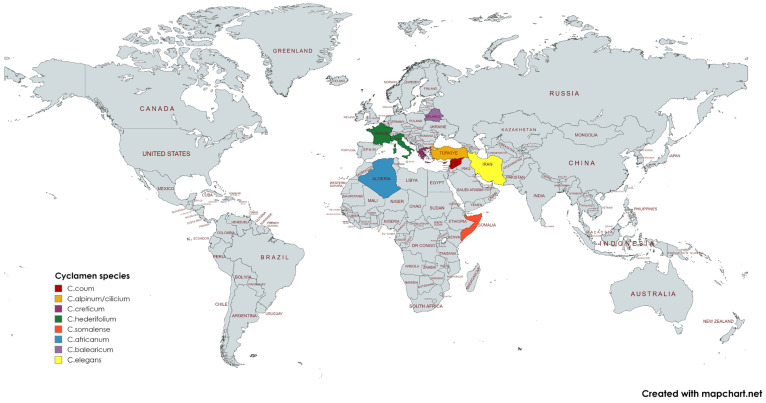
Distribution map of *Cyclamen* species in Europe, Asia, and Africa [21]. Created with MapChart (https://www.mapchart.net/world.html, accessed online on 21 June 2024).

The distribution of *C. hederifolium* has expanded into the southern United Kingdom and is widely distributed throughout Europe [21]. It is native to Greece, western Turkey, southern France, and Italy. It is resistant to cold and heat with significant intraspecific variation. One of the most common examples of distant cross-breeding of *Cyclamen* uses *C. hederifolium* as the parent crossed with *C. persicum* [24]. In numerous Serbian locations, *C. hederifolium* has been used as a purgative, anticancer treatment, and treatment for rheumatism, skin disorders, menstrual pains, and migraines [25]. Small flowers with a wide range of leaf colors, including white-green stripes, light green, and green-white center, characterize *Cyclamen hederifolium*. Although the white and green striped variant of leaves is unique and has significant artistic value, its production method is yet unclear [26]. *C. libanoticum* is endemic to the Lebanese flora [27]. The geographic distribution of various *Cyclamen* species is shown in Figure 2.

## 3. *Cyclamen* Composition

### 3.1. Cyclamen Somatic Embryogenesis

*C.coum* Every species of *Cyclamen* has a specific month and season of growth based on its characteristics and locality, as shown in Table 1. Somatic embryogenesis is a biotechnological technique primarily applied to the clonal propagation of various plants all over the world. In somatic embryogenesis, under the right culture conditions, embryos develop from somatic cells. It is the best strategy among the genetic transformation protocols used for plant regeneration and is crucial for true cellular totipotency [28]. This process is divided into different phases with different conditions. First, the best explant type for *Cyclamen* is ovules from closed, unpollinated flower buds, because these explants are found on mature plants that have been assessed for their breeding or commercial value, they can be harvested without damaging the plant, and importantly hardly ever result in contaminations in tissue culture [29]. The second step is called the realization phase, where one of the two cotyledons is suppressed very early, resulting in the development of just one cotyledon in both zygotic and somatic embryos [30]. In the last step, a mature somatic embryo develops into a small plantlet that can adapt to light and be planted in soil [31].

### 3.2. Color Distribution among Different Parts of Cyclamen Leaves and Flowers

**Figure 3:**
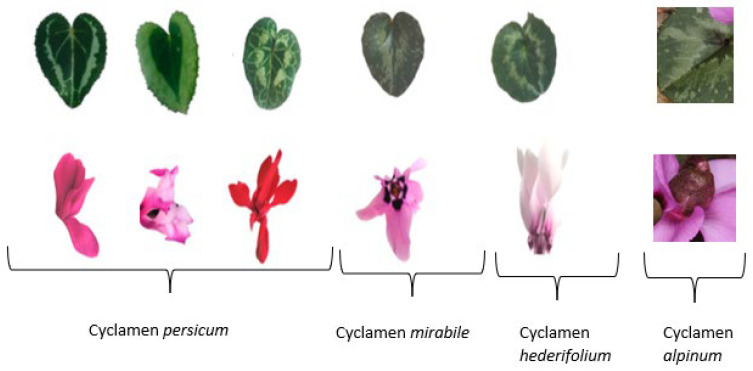
The different sizes and shapes of leaves and flowers of various *Cyclamen* species.

*Cyclamen* flowers are of different colors, shapes, and sizes as shown in Figure 3. They are responsible for attracting pollinators like insects, enhancing fruit production. There are multiple pigments responsible for the enormous variety of flower colors [32]. The primary pigments found in *Cyclamen* are flavonoids, carotenoids, chlorophylls, and betalains [33]. Among the flavonoids, anthocyanins are red to purple, and flavonol glycosides are colorless or pale compounds. In the *Cyclamen* flower, flavonyl glycosides act as co-pigments, altering the anthocyanin colors and producing a variety of colors [34].

Based on high-performance liquid chromatography-photo diode array (HPLC-PDA) analysis, five individual carotenoids including neoxanthin, violaxanthin, lutein, β-carotene, and cis-β-carotene were observed as the main components in *Cyclamen* leaves [25]. Seven individual anthocyanins, which are water-soluble pigments, have a broad assortment of colors, such as orange, pink, red, blue, and purple hues, based on the environmental pH. These anthocyanins, including cyanidin 3,5-di-*O*-glucoside, peonidin-rutinoside, peonidin 3,5-di-*O*-glucoside, peonidin 3-*O*-glucoside, malvidin 3-*O*-glucoside, malvidin 3,5-di-*O*-glucoside, and malvidin-rutinoside are main components in *Cyclamen* flowers [35]. Numerous factors can impact the color of the flower. The first is pH, where anthocyanins show different colors at different pHs. Also, complexing with different ions like Al^3+^ and Mg^2+^, and interacting with other colorless molecules, such as co-pigments can affect the color [36].

### 3.3. Sugar Content

Total sugar distribution throughout a plant is correlated with the flux of carbon, osmolytes, and energy during growth [37]. Additionally, proline and soluble carbohydrates are essential for plants to respond to stress [38]. The soluble sugar concentration of the entire plant was enhanced throughout the seasonal co-existence of leaves and flowers, and it gradually decreased as the above-ground plant parts shed. Pedicels and petals have less sugar in them than leaves and tubers do. From late autumn to early spring, the starch content of leaves increased and was comparable to that of tubers [39]. In contrast to the rest of the year, proline concentration in *C*. *graecum* tubers increased little, but statistically significantly from November to March. This buildup might be caused by the chilly months in question and might be seen as a stress-related reaction [40]. It has been documented that the species *C. graecum* is cold [41]. The protective function of proline, which was favorably connected with the turgor of *C. graecum* petals, has also been emphasized during floral developmental processes that include dryness, as naturally happens during pollen production or embryogenesis [42]. Proline may additionally function as a practical supply of energy and nitrogen during the immediate post-stress metabolism [43].

### 3.4. Flower Fragrance and Volatile Compounds

**Figure 4:**
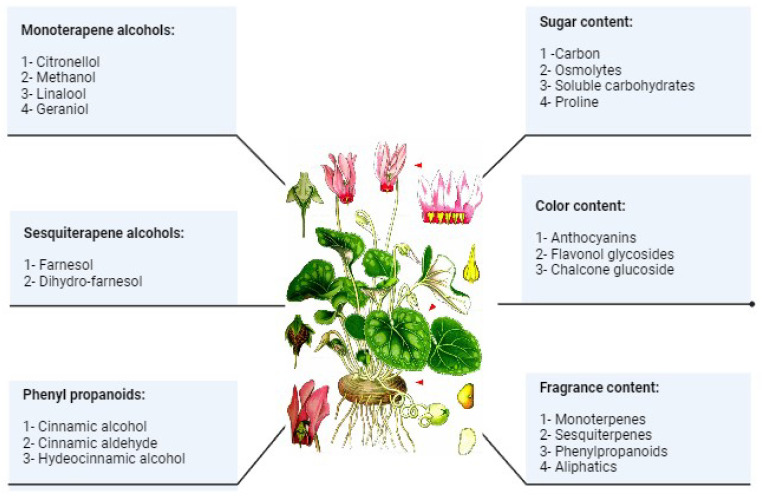
The main volatile compounds, sugar color, and fragrance content that are produced by *Cyclamen purpurascens* mill.

In flowers, leaves, and fruits, more than 1700 low molecular weight volatile compounds have been discovered (Figure 4). They are classified as terpenoids, phenylpropanoids/benzenoids, aliphatic, nitrogen, sulfur, and other cyclic molecules with a variety of functional groups, such as acids, aldehydes, ketones, alcohols, esters, and ethers [44]. These molecules are produced in unspecialized epidermal cells of floral organs, particularly petals, or specialized gland cells on the surface of leaves and stems. They draw pollinators, prevent herbivorous insects, and draw predatory insects naturally [45].

## 4. Bioactive Potential Efficiency of *Cyclamen* Plant

### 4.1. Antioxidant Activity

Antioxidants are substances that prevent molecules from being harmed by free radical-induced oxidant chain reactions from starting or continuing. Natural and artificial antioxidants are separated [46]. Antioxidants have a strong biological activity in reducing the effect of reactive oxygen species (ROS) produced by human body mechanisms including respiration, metabolizing therapeutic agents, digesting food, and converting fats into energy [47]. Also, by transferring electrons to free radicals and preventing the oxidative process, antioxidants can effectively block or slow down the interactions of biomolecules with free radicals, which can be useful in the suppression and/or treatment of chronic illnesses [48]. Due to their robust biological activity, synthetic antioxidants used in the industry are more likely to have side effects and act as cancer-causing catalysts [49]. Synthetic antioxidants like butylated hydroxytoluene (BHT) which is a phenolic antioxidant tested on different strains of mice and shown to induce lung injury and cancer [50]. Moreover, butylated hydroxyl anisole (BHA) has been added to food as an antioxidant for the past century [51]. The use of this synthetic molecule has been limited, though, as it has been linked to potential toxicity and has been known to have certain side effects, including carcinogenesis [52]. Natural plant extracts have the potential to serve as substitutes for artificial preservatives, such as antioxidants, while also adding bioactive qualities and value to the finished goods [22].

Due to this, the need for natural antioxidants has started to become more crucial, and in recent years, the search for plants that naturally contain antioxidants has become more important [53]. Ascorbic acid is one of the most extensively studied antioxidant compounds in plant cells [54]. It is involved in cell expansion [55], cell division defense, and growth [56]. Ascorbate peroxidase is one of the numerous enzymes that ascorbic acid cofactors [57]. Ascorbic acid is the primary reducing substrate for H_2_O_2_ removal in plant cells [58].

Antioxidants that can neutralize free radicals may therefore play a role in preventing illnesses including cancer, cataracts, aging, etc. [59]. It was found that the tuber part of *Cyclamen* was more responsive than the leaf part and demonstrated more ability to act as a free radical scavenger [60]. *Cyclamen alpinum’s* phenolic and flavonoid content has the highest antioxidant potential assessed by a variety of analytical techniques, like the 2,2-diphenyl-1-picrylhydrazyl (DPPH) test [61,62]. The investigations of *Cyclamen* plants indicated an improvement in the antioxidative activity that is produced and occurs by mycorrhizal symbiosis. Under biotic stress, increased DPPH radical scavenging activity has also been seen in mycorrhizal asparagus [63].

### 4.2. Anti-Inflammatory Effect

In vascular tissues, inflammation is a part of the complex biological reaction to pathogens, injured cells, or irritants [59]. However, we typically always experience inflammation in conjunction with an infection, even though an infection does not always accompany inflammation [64]. Numerous plant extracts interact with different pathways involved in the biosynthesis of inflammation mediators to provide therapeutic effects. Antioxidant power is often exhibited by natural active principles with anti-inflammatory activity [65]. For this reason, it seemed of interest to check extracts of *Cyclamen* for antioxidant capacity, using two different in vitro chemical methods: the Briggs–Rauscher reaction method [66] and the trolox equivalent antioxidant capacity (TEAC) assay [67].

Many triterpenoids have been shown to have anti-inflammatory properties via a variety of mechanisms, such as the inhibition of cytokine and eicosanoid production, lipid peroxidation, hydrolytic enzyme activity, and interactions with certain serine/threonine kinase harmful agents such as pathogens, injured cells, or irritants [68]. The findings support the anti-inflammatory properties of *Cyclamen* and imply that the triterpenic saponin structures are involved in the therapeutic benefit. Even though it has not been noted in the plant’s traditional uses, this type of activity can be linked to the isolated triterpenic compounds for other therapeutic purposes [69].

In herbal medicine, the tuber of *Cyclamen europaeum* (*Cyclamen purpurascens*), a plant belonging to the *Primulaceae* family, has been used for a variety of purposes in the form of nasal sprays [70]. The frozen, dried, natural fluid extract of the *Cyclamen europaeum* plant that is delivered intranasally has beneficial effects in relieving congestion by facilitating nasal drainage in addition to its anti-inflammatory effect [71]. Patients’ symptoms, nasal endoscopic signs, and satisfaction were significantly improved after receiving *Cyclamen europaeum* extract instead of saline during endoscopic sinus surgery in patients with chronic sinusitis and nasal polyps. These outcomes are believed to be related to the extract’s ability to promote nasal drainage and clear the paranasal sinuses [72].

### 4.3. Anti-Cancerous Effect

Cancer is one of the main causes of mortality worldwide [73]. A variety of temporal and spatial physiologic changes in cancer cells eventually result in malignant tumors [74]. The bulk of these tumors may be stopped by our body from drawing in fresh blood, preventing their potential growth caused by a deficiency in nutrients and oxygen. So, an in-situ tumor can stay latent indefinitely if there is no angiogenesis which is the growth of new blood vessels [75]. Environmental exposures rather than genetic predispositions were the primary cause of the majority of cancers [76]. The majority of cancer types are caused by chronic inflammation, for example, esophageal cancer, which is typically preceded by years of inflammation caused by gastroesophageal reflux, lung cancer, where smoking usually results in inflammation, and colon cancer, which is occasionally linked to chronic inflammatory bowel disease. This was shown to be due to a microbial presence in or near the epithelia stimulating the recruitment and activation of inflammatory cells stimulating the recruitment and activation of inflammatory cells. This activation leads to a respiratory burst that releases free radicals. By peroxiding lipids and causing genetic mutations, which can change proteins by chemical and post-translational modifications, free radicals contribute to malignant transformation; such damage to epithelial cells triggers reactive epithelial hyperproliferation and apoptotic cell death, which encourages additional mutation [77].

Traditional medicine uses *Cyclamen persicum* to cure a variety of illnesses, including anti-rheumatic, diarrhea, abdominal aches, edema, abscesses, eczema, and cancer [78]. *C. persicum* had a substantial cytotoxic effect on the PC-3, MCF-7, and LNCaP cell lines, inhibiting them by more than 90% at a concentration of 0.5 mg/mL. The antioxidant properties of this species have been previously evaluated, which suggested the possibility of anti-proliferative potential for these plants [78]. Moreover, *Cyclamen pseudibericum* (CP) extracts showed cancer invasion and migration inhibitory effect by associating zinc-finger E-box binding homeobox 1 (ZEB1) mediated epithelial to mesenchymal transition (EMT) [79]. Zinc finger E-box binding homeobox 1 (ZEB1) is a transcriptional repressor that regulates the epithelial-to-mesenchymal transition (EMT), which can be triggered by a variety of invasion inducers [80].

**Figure 5:**
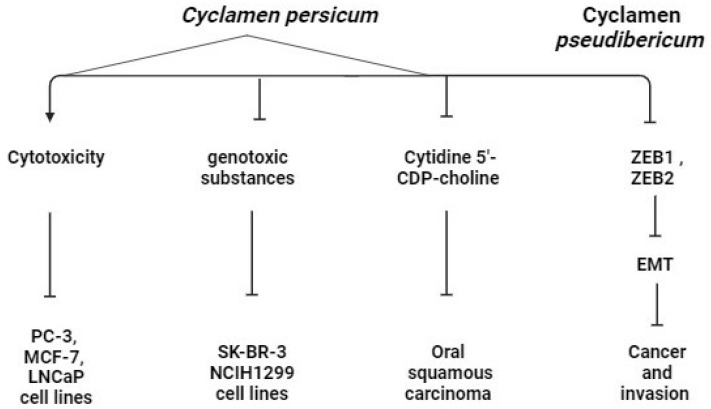
The anti-cancerous pathway of *Cyclamen* in different cancer cell lines. (PC-3: prostatic adenocarcinoma cell line, MCF-7: human breast cancer cell line, LNCaP: prostatic adenocarcinoma cell line, SK-BR-3: breast adenocarcinoma, NCIH1299: lung adenocarcinoma, ZEB: zinc finger E-box binding homeobox, EMT: epithelial-to-mesenchymal transition).

High levels of ZEB1 drive the progression of certain cancers, including ovarian, breast, pancreatic, liver, and lung cancers [81,82]. By reducing the expression of their target genes, miRNAs have been shown to control either a single stage or many steps of metastasis [83]. The transcriptional repressors ZEB1 and ZEB2 are inhibited by the miR-200 family of miRNAs in epithelial cells, and the suppression of these genes is crucial in preventing associated factors from initiating the EMT (Figure 5) [84]. By using qRT-PCR, it was demonstrated that the expression of miR-200c was significantly lower in NSCLC cells as compared to normal lung epithelial cells. In addition, the overexpression of miR-200c in A549 cells increased the expression of E-cadherin and decreased that of N-cadherin and vimentin. Consequently, lower ZEB1 expression in the A549 cell line was linked to members of the miR-200 family [85].

Saxifragifolin B is a triterpenoid tetrasaccharidic saponin. It has been previously isolated from different plants, mainly Cyclamen, like *Cyclamen persicum* and *libanoticum* [27]. Cyclamin is a triterpenoid pentasaccharidic saponin. It has been previously isolated from other *Cyclamen* against breast adenocarcinoma (SK-BR-3) and lung carcinoma (NCIH1299), so Saxifragifolin B has the potential to be a cytotoxic drug with a preventive effect against exposure to genotoxic substances in the environment and during chemotherapy. It could be prescribed as a complementary medicine to enhance the anticancer effects of traditional chemotherapeutic agents and to reduce their side effects [86]. Tests were done on cyclamin and saxifragifolin to study their anticlastogenic effect using mitomycin C as a DNA-damaging agent. So, this bifunctional alkylating compound is a potent DNA cross-linker, specific for a guanine nucleoside. It induces DNA damage leading to deletion mutations [87] and chromosome abnormalities [88].

About 1% of all reported neoplasms and 12–15% of hematological malignancies are malignant plasma cell disorders called multiple myeloma (MM), which can lead to hypercalcemia, bone disease, anemia, and renal failure [89]. Isoprenoids are one class of phytochemicals that play an essential role in the negative regulation of cell proliferation, apoptosis, and differentiation [90,91]. One such isoprenoid, farnesol (FOH), is present in many essential oils such as *Cyclamen* [92] and used to treat obesity, diabetes, hyperlipidemia, and atherosclerosis [93]. FOH has been shown to antagonize the Cytidine 5′-CDP-choline pathway [94] and exhibits anticancer potential as indicated by its ability to decrease the proliferation of a wide variety of human tumor cell types including oral squamous carcinoma [95]. The biological impact of different *Cyclamen* species on cancer cell lines is presented in Table 2.

## 5. Therapeutic Applications and Clinical Trials of Cyclamen

In clinical practice, rhinosinusitis, an inflammatory condition of the paranasal sinuses, is very common [100]. An inflammatory change of the nose and paranasal sinuses is known as acute rhinosinusitis (ARS). Although a viral infection is the typical cause of ARS, other conditions such as allergic rhinitis, pathological abnormalities, nasal polyps, and abusing nasal decongestants may serve as risk factors [101]. *Cyclamen europaeum* (CE) is a safe and effective treatment that significantly reduced the severity of each patient’s symptoms (including nasal obstruction, mucus secretion, facial pain, and loss of smell), improved mucosal edema and nasal obstruction as determined by endoscopy, and decreased sinus occlusion as determined by CT scan on day seven [102]. After 3 to 5 days of treatment, CE begins to affect ARS, and after 9 to 12 days, all symptoms disappear completely. Comparing CE to antibiotic treatment, patients and researchers gave CE much higher satisfaction ratings in terms of reducing the time it took for the disease to progress, the need for antibiotics or enhancing their effects, the number of problems, and the chronification of the disease [103].

Additionally, recent research assessed the effectiveness and safety of CE and concluded that it offers a good choice for treating ARS, ensuring individualized care, and reducing polymerization and improper antibiotic use [104]. Therefore, when added to other combinations or used as a monotherapy in comparison to other monotherapies, the usage of CE was linked to a better cure rate without raising medical costs. It is crucial to remember that untreated rhinosinusitis may cause a recurrence or even a potential chronification of the illness, which would lower the patient’s general health and quality of life, and raise direct and indirect costs [105].

A recent study conducted in vivo using a therapeutic equivalent dose resulted in a mucosa secretion that lasted for about 15 min and showed no symptoms of irritation or itching, attesting to the adequate safety profile of *Cyclamen* lyophilized extract [106]. Moreover, an actual observational study showed that *Cyclamen europaeum* reduced the impact of oral antibiotics on exacerbations and recurrences of chronic rhinosinusitis. Compared to antibiotics in monotherapy, CE extract nasal spray significantly reduced sinonasal symptoms and CRS recurrences in patients with moderately severe CRS exacerbations when used as a monotherapy or in addition to standard antibiotic treatment [107]. Triterpene glycosides, also known as saponins, are the primary biologically active components of *Cyclamen* and are distinguished by their strong surfactant qualities. The vestibule and anterior regions of the inferior concha of the nose are the only areas where saponins initially act. Here, they stimulate the nociceptive endings of the ethmoidal nerve, a branch of the trigeminus nerve. Consequently, this elicits secretory and other reflex responses. The elimination of the response with a local anesthetic spray containing 1% dicaine before the instillation of saponins further supports the reflex nature of the response. As surface-active compounds function as detergents to lower surface tension on cell membranes, a physical action mechanism for saponins has also been described. This effect is the basis for the hemolytic effect of saponins that have been described. When used to treat RS symptoms, it may also be the cause of the flushing effect that releases accumulated mucus in the sinuses [98,108,109].

Numerous clinical trials have been conducted to evaluate the effectiveness of herbal extracts in patients with RS. When compared to a placebo, *Cyclamen* saponins freeze-dried extract lessens the symptoms of acute RS, restores mucociliary movements, and increases the rate of remission. The treatment group experienced a greater number of side effects, primarily from localized irritation at the treatment site. Compared to placebo treatment, where 24% of participants reported mild events, 50% of clinical trial participants experienced mild events like sneezing and irritation of the nose and throat. No serious side effects were reported. Additional clinical trials evaluating *Cyclamen* saponins in acute RS in conjunction with antibiotics and in chronic RS have been conducted, yielding positive outcomes when compared to monotherapy or combination therapies [71,105,107,110].

Moreover, the impact of topical intranasal *Cyclamen europaeum* extract on the clinical reaction in children and adults suffering from acute sinusitis has been evaluated. Randomized controlled trials were used to compare the intranasal administration of *Cyclamen europaeum* extract to no treatment, intranasal corticosteroids, antibiotics, or placebo for individuals suffering from acute sinusitis in adults, children, or both. In two randomized controlled trials, 147 adult outpatients with acute sinusitis confirmed by nasal endoscopy or radiology were divided into study groups and given either *Cyclamen europaeum* nasal spray or a placebo for a maximum of 15 days. There were no recorded major side effects or treatment-related complications, but participants in the *Cyclamen europaeum* group experienced more mild side effects, such as sneezing, mild epistaxis, and nasal and throat irritation at 50% compared to participants in the placebo group (24%); this difference was supported by moderate-quality evidence (risk ratio 2.11, 95% confidence interval 1.35 to 3.29) [71]. Table 3 illustrates the main active components and mechanism of action of the *Cyclamen* plant responsible for its therapeutic effect.

## 6. Conclusions and Future Perspectives

*Cyclamen* is a perennial winter-blooming flowering plant with plenty of potential bioactivities. *Cyclamen* is a plant with a long history of use in medicine that has pharmaceutical, medicinal, and nutraceutical qualities. This is explained by the abundance of polyphenolic compounds and flavonoids, which are significant secondary metabolites with a wide range of biological activities. The antioxidant activity and other biological activities displayed by *Cyclamen* are facilitated by the presence of flavonoids and polyphenolic substances. This implies that it may have the ability to act as a natural antimicrobial and a natural source of anticancer compounds. These results underline the potential for more research on *Cyclamen* as a source of therapeutic agents while also supporting its traditional medical use. This review also focuses on the diversity of *Cyclamen* species, its chemical composition, coloring content, its stages of development, and different localizations of the species. In addition to that, this review sheds light on the potential antioxidant activities of *Cyclamen* accompanied with its anti-inflammatory effects and imposes the activity of *Cyclamen* extracts as anti-cancerous compounds against various cell lines. Moreover, *Cyclamen* has proven its efficiency in some therapeutic cases like sinonasal cases and acute rhinosinusitis, and paves the road towards future research based on *Cyclamen* extracts against other related medical conditions.

Moreover, future research endeavors must focus on examining the impacts of distinct *Cyclamen* sections with the isolation and identification of the compounds accountable for the anticancer properties of the solvent extracts. Additional research is needed to isolate and identify the active components as well as thoroughly investigate the molecular mechanism underlying the bioactivities of this plant which will be useful in the creation and advancement of natural anticancer medications, as well as informing potential future research projects in this field. *Cyclamen* has been the subject of few cytotoxic studies. When treating non-complicated, non-severe acute exacerbation of chronic rhinosinusitis, intranasal *Cyclamen* extract may be an alternative to standard antibiotic therapy. The plant extract also demonstrates mild inhibitory activity against both Gram-positive and Gram-negative bacterial strains. Furthermore, the literature did not contain any in vivo studies that described the toxicological profile of *Cyclamen* saponins after nasal administration. It should be mentioned that more research is required on the isolated chemical compounds to determine other pharmacological effects and potentially identify additional biologically active substances. The data in this study can theoretically support the viability of additional pharmacognostic research on this plant, including the creation of pharmacopoeial monographs and techniques for quality control for unprocessed herbal drugs to incorporate products derived from it into mainstream medicine. It may also help lower disease costs and prevent antibiotic abuse, which in turn lowers the rise in antibiotic resistance.

## Figures and Tables

**Table 1:** Seasonal variations and growth of various *Cyclamen* species. The green color indicates the growth month, and the blue color indicates the growth season.

	*C. alpinum*		*C. cilicium*		*C. coum*		*C. criticum*		*C. hederif-olium*		*C. mirable*		
December													
November													
October													
September													
August													
July													
June													
May													
April													Summer
March													Spring
February													Winter
January													Autumn

**Table 2:** Biological and cancer cell line effects of various Cyclamen species. (↑: increases, ↓: decreases).

Cyclamen Species	Plant Part	Biological Pathway Effect	Cancer Cell Line Effect	Reference
*C. alpinum* Dammann ex. spreng	Tubers	↑ toxic LC50 in T. tubifex	↓ cytotoxicity in HCT 116 and HT29	[60,96]
*C. coum Mill*	Multiple parts	↓ antibacterial activity	↑ cytotoxicity HeLa and NSCLC H1299	[82,97]
*C. hederifolium* Aiton	Tubers	↓ cytotoxic activity	↓ apoptosis in Hela, H-446, HT-29, U937	[98]
*C. persicum* Mill	Tubers	↑ cytotoxic activity	↑ apoptosis in human carcinoma nasopharynx	[99]
*C. pseudibericum* Hildebr.	Tubers	↓ cytotoxic activity	↑ cytotoxicity on A549 cells ↓ number of A549 cells	[79]

**Table 3:** The mechanism of action of the main active component responsible for the therapeutic effect of various *Cyclamen* species.

Species	Active Component	Therapeutic Effect	Mechanism of Action	Reference
*Cyclamen persicum*	Saponins (repanoside)	Anti-inflammatory	Stimulate nasal receptors Release inflammatory sinus exudates Relieves congestion	[111]
*Cyclamen persicum*	Flavonoids polyphenols	Antioxidant	Free radical scavengers hydrogen donors, reducing agents, and singlet oxygen quenchers	[111]
*Cyclamen europaeum*	Tannins Carotenoids (lutein and β-carotene)	Anti-cancerous	Anticancer effect on MCF7 breast cancer and colorectal adenocarcinoma (HT29) colon cancer	[111]
*Cyclamen europaeum*	Anthocyanins (Malvidin 3-*O*-glucoside cyanidin 3,5-di-*O*-glucoside) with values ranging from 40.5% to 75.7%	Anti-cancerous	Inhibitory activities against the cell proliferation of stomach, colon, lung, breast, and CNS cancer cells	[25]
*Cyclamen repandum*	Triterpenoids Polyphenols Flavonoids Saponins	Antioxidant Anti-inflammatory	Inhibit eicosanoid and cytokine production, hydrolytic enzyme activity, lipid peroxidation, and interaction with some serine/threonine kinases	[69]
* C. europaeum *	Triterpene saponins	Anti-inflammatory	Secretory Drainage stimulation Mucosa secretion Sufficient safety profile	[106]
* C. coum *	Saponins Phenolic compounds	Antifungal	The interaction of aglycone fragments of saponins and fungal membrane sterols is the main mechanism that causes the formation of transmembrane pores, it destroys the integrity and leads to membrane lysis.	[112]
* C. coum *	n-butanolic extract	Antibacterial Antibiofilm	Prevents the *Pseudomonas aeruginosa* biofilm from growing, which is a significant factor for individuals with cystic fibrosis.	[6]
*C. trochopteranthum*	Water extract	Anti-cancerous	Increases CYP1A1/2 mRNA level Decreases CYP2B6 mRNA level Human hepatocellular liver carcinoma cell line (HepG2) and human epithelial colorectal adenocarcinoma (Caco2).	[113]

## Data Availability

This paper has all the data supporting the findings.
